# Imperative for a health‐centred focus on climate change in radiology

**DOI:** 10.1111/1754-9485.13813

**Published:** 2024-12-11

**Authors:** Omar Taboun, Chloe DesRoche, Kate Hanneman

**Affiliations:** ^1^ Schulich School of Medicine & Dentistry Western University London Ontario Canada; ^2^ Department of Diagnostic Radiology Queens University Kingston Ontario Canada; ^3^ Department of Medical Imaging University of Toronto Toronto Ontario Canada; ^4^ Joint Department of Medical Imaging University Medical Imaging Toronto, University Health Network (UHN) and Sinai Health System (SHS) Toronto Ontario Canada

**Keywords:** quality assurance

## Abstract

Climate change negatively impacts individual and population‐level health through multiple pathways, including poor air quality, extreme heat and changes in infectious disease. These health effects will lead to higher health system and medical imaging utilisation. At the same time, the delivery of radiology services generates substantial greenhouse gas emissions. Mitigation strategies to reduce the environmental impact of medical imaging and adaptation strategies to build resiliency to current and future impacts of climate change in radiology should be centred on human health. A health‐centred response in radiology reinforces the role of radiologists as physicians and emphasises the opportunity for medical imaging to promote health and advance our understanding of climate‐related health effects. This review discusses the need for a health‐centred focus on climate change in radiology, including the effects of climate change on human health and health systems, intersection of climate change with health equity, health benefits of climate action and opportunities to leverage medical imaging to improve human health.

## Introduction

After the industrial revolution, we entered a new era referred to as the Anthropocene Epoch—an unofficial unit of geologic time defined by the start of human activity having a significant impact on the planet.^1^ The planetary health framework emerged in this context to acknowledge and address the link between human activities and the consequential biophysical change it brings to the planet.^2^ This change exists within many dimensions, such as pollution of water, air and soil, disruption of global climate systems (climate change), biodiversity loss and resource depletion among others. Planetary health also strives to recognise and address how these fundamental changes to the planet impact human health.^3^

Health care is essential to respond to the adverse health impacts of climate change. At the same time, the delivery of healthcare generates substantial greenhouse gas emissions, thus contributing to the climate crisis. The overall healthcare sector is estimated to contribute between 4% and 8% of total global greenhouse gas emissions with estimates varying depending on the country and health system.^4^ Within healthcare, hospitals are responsible for generating between 31% and 37% of total greenhouse gas emissions.^5^ Healthcare‐related emissions include direct emissions from the operation of healthcare facilities (scope 1) as well as indirect emissions from purchased sources of energy (scope 2) and the entire supply chain of related goods and services (scope 3). Medical imaging contributes an outsized share of these emissions, largely driven by the energy intensity of medical imaging equipment.^6^, ^7^ Beyond the generation of greenhouse gas emissions, the negative environmental impact of the delivery of radiology care extends to include depletion of finite resources such as helium, contamination of waterbodies related to gadolinium‐ and iodine‐based contrast media and generation of medical and non‐medical waste.^8^, ^9^

**Fig. 1:**
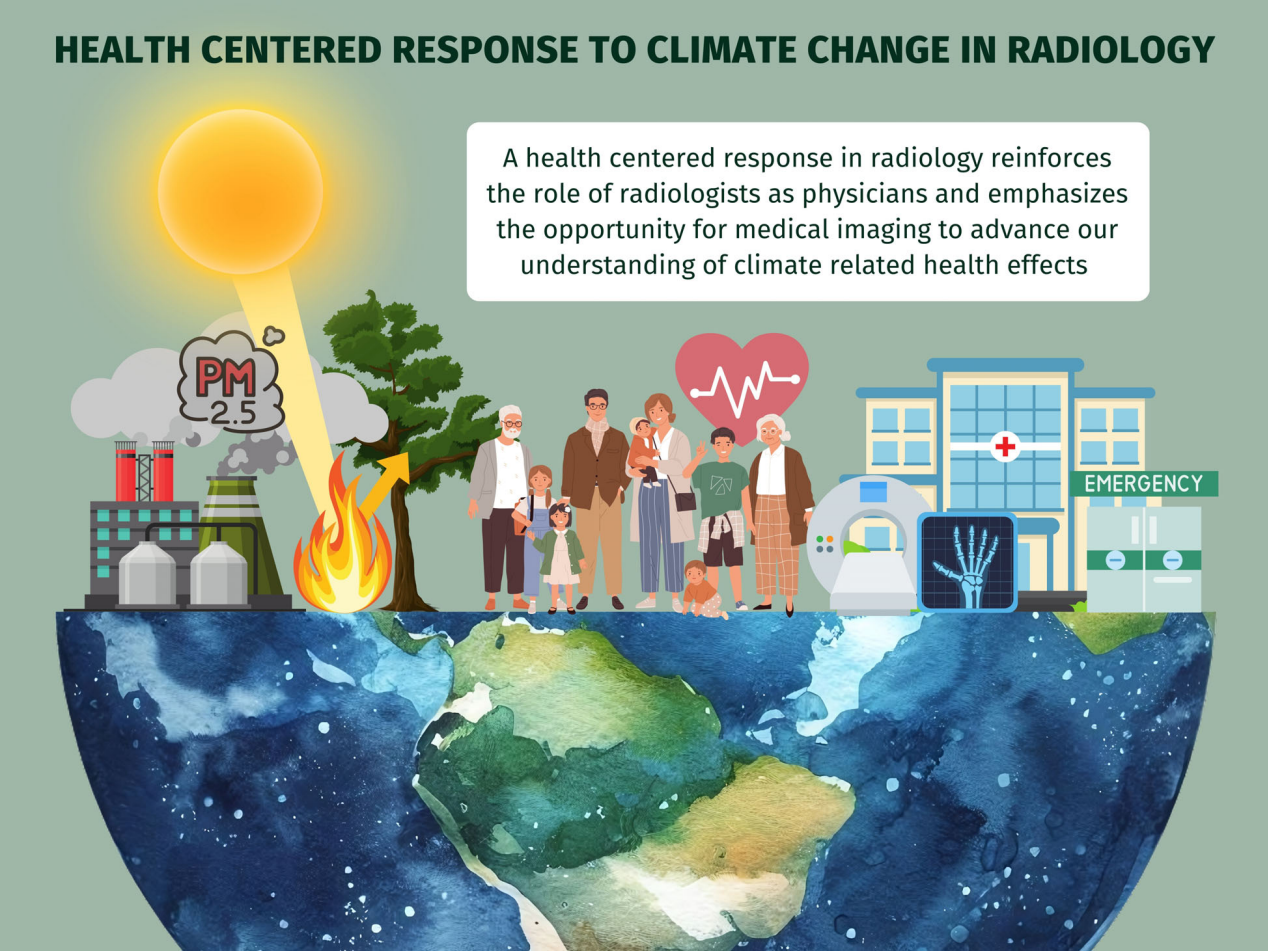
A health‐centred response to climate change is needed in radiology.

To date, the climate and sustainability‐related literature in radiology has largely focused on the environmental impact of delivering radiology services and the need for mitigation strategies to reduce greenhouse gas emissions and waste.^6^, ^8^, ^9^, ^10^, ^11^, ^12^, ^13^ However, there is growing recognition that adaptation strategies are also needed to build resiliency to the current and future impacts of climate change.^14^ Sustainable radiology should be centred on human health.^15^ Focussing exclusively on electricity, power or energy savings may not resonate with key stakeholders outside of radiology. However, a health‐centred response in radiology reinforces the role of radiologists as physicians and shifts the framing of sustainable radiology from *radiology as a problem* to *radiology as part of the solution*, Figure 1. In this review, we discuss the need for a health‐centred focus on climate change in radiology including the effects of climate change on human health and health systems, intersection with health equity, health benefits of climate action and opportunities to leverage imaging to improve human and planetary health.

## Health impacts of climate change

Climate influences all life on earth and plays a role in determining the boundaries in which species can adequately survive and reproduce. Human health is impacted by changes in temperature, precipitation, weather, air quality, water quality and food.^16^

### Biodiversity loss

Climate change, biodiversity loss and pollution are part of the interlinked triple planetary health crisis. Biodiversity refers to the variety of life on Earth, in all its forms. Climate change is a primary driver of biodiversity loss, related to changing temperatures and precipitation patterns.^17^ Countries with less wealth have greater biodiversity loss, highlighting health inequities of planetary health. People living near more green or natural environments with more biodiversity—such as forests, parks and coastal regions—have better health outcomes compared to those living in urban, less biodiverse, environments.^18^ The positive health outcomes associated with living near natural environments with higher biodiversity include psychological well‐being, such as increased attention, decreased depression and stress, as well as physiological outcomes, such as reduced blood pressure, reduced general illness and reduced obesity. Biodiversity underpins ecosystem functioning and the provision of food and other essentials for human health and well‐being. Therefore, biodiversity loss can have significant direct human health impacts if ecosystems are no longer adequate to meet social needs.

### Air pollution

Air pollution is the leading environmental health hazard contributing to excess morbidity and mortality. Air quality is impacted by multiple factors but is closely linked to climate change. Air pollutants are frequently emitted along with climate change causing greenhouse gases due to human activities such as burning fossil fuels. Climate change has also increased the frequency and severity of wildfires which negatively impact air quality due to smoke. Although air quality has improved over the past few decades due to increased regulations, it is still ubiquitous. Over 99% of the world's population lives in places where World Health Organization air quality guideline levels are not met.^19^ Over 4 million deaths were attributed to ambient air pollution in 2019.^20^ Air pollution impacts the neurovascular, cardiac and respiratory systems with adverse outcomes including myocardial infarctions, arrhythmias, peripheral artery disease and asthma exacerbations.^21^ Short‐ and long‐term exposure to fine particulate matter air pollution is associated with increased frequency of cardiovascular risk factors including hypertension and atherosclerotic disease.^22^, ^23^ In the short term, acute cardiovascular and respiratory presentations related to air pollution exposure increase health system access and imaging utilisation. Medical imaging has an important role in investigating chronic effects of longer‐term air pollution exposure, such as coronary CT for evaluation of atherosclerotic disease.

### Heat exposure

Increasing global temperatures and exposure to extreme heat have both short‐ and long‐term adverse health impacts. A recent meta‐analysis found that heatwaves increased mortality by 18% and morbidity by 10% in patients with diabetes.^24^ Extreme temperatures are also associated with worse respiratory, cardiovascular and neurological outcomes and higher healthcare use.^16^ High heat stress can also reduce physical work capacity and cognitive performances, with consequences for productivity and increased risk of occupational health injury.^25^ Heat‐related morbidity and mortality are projected to increase further as climate change progresses. In cities, ambient temperatures are further exacerbated by anthropogenic heat from vehicles and heat waste from buildings.

### Changing disease patterns

Epidemiological shifts in disease transmission patterns are directly influenced by climate change. Changing disease patterns directly impact the pathology and disease processes visualised on medical imaging. Diseases that were previously rare will become endemic or more prevalent in new areas due to an expanded range of vector‐borne and infectious disease. A recent study estimated that 58% of the infectious diseases have been aggravated by climate change.^26^ Changes in temperature, rainfall and humidity impact the transmission of many mosquito‐borne diseases including dengue, malaria, yellow fever, West Nile fever and Zika among others.^27^ As summers get longer and warmer, there has been a dramatic increase in dengue from 500,00 cases in 2000 to 5.2 million in 2019, and the expansion to geographic regions previously considered low risk such as the US and Europe.^27^ Additionally, climate change is associated with an increase in antibiotic‐resistant infections. A temperature rise of 10°C across geographic regions was associated with increased antibiotic resistance for common pathogens such as *Escherichia coli* (4.2%), *Klebsiella pneumonia* (2.2%) and *Staphylococcus aureus* (2.7%).^28^ This could potentially impact the frequency and severity of infectious disease processes on imaging, such as pneumonia.

Over the past several years, health care has faced major challenges related to infectious disease outbreaks including Ebola, monkeypox, measles and COVID‐19. Recognition of new and emerging or re‐emerging diseases related to climate change will pose diagnostic challenges and impact imaging findings and volumes.^29^ Adaptation planning in radiology can build on protocols and policies developed for pandemic planning preparedness.

## Impact on medical imaging delivery

Health systems are increasingly strained to provide care for growing and aging populations. The ability to deliver healthcare—including radiology services—is also impacted by climate change including increased utilisation, more severe and frequent disease and exacerbation of pre‐existing disease. Increased healthcare needs will increase utilisation of an already taxed healthcare system and will also result in higher costs and longer wait times. This is a global challenge with 27% of cities surveyed reporting concerns over their health systems being overwhelmed by the impacts of climate change.^15^

Increases in ambient temperature are associated with increased emergency department visits and hospital admissions. A study of 13 hospitals in Perth, Western Australia, found that 4.6% of total emergency department visits were attributable to heat, resulting in AU$2.9 million in excess healthcare costs.^30^ In the US, climate change alone is estimated to result in an additional 21,000–28,000 hyperthermia‐related emergency department visits, with associated treatment costs estimated between $6 million and $52 million by 2050.^31^

Poor air quality is also associated with higher health system use. A 10 ug/m^3^ increase in daily wildfire‐related fine particulate matter (PM_2.5_) concentration was associated with a 3% increase in intensive care unit admissions 5 days later for asthma exacerbations.^32^ Wildfire‐related smoke and air pollution are also associated with a 2% increase in emergency department visits for respiratory conditions on the day of exposure.^33^

Higher emergency department visits and hospital admissions due to climate change‐related environmental exposures result in higher imaging volumes. In an analysis of 1,666,420 medical imaging studies from four emergency departments over a 10‐year period, short‐term exposure to higher ambient temperature and PM_2.5_ air pollution was associated with overall imaging utilisation increases of 5.1% and 4.0%, respectively.^34^ The demand for imaging services has substantially increased over the last decade and continues to rise.^35^ In parallel, increased emergency department visits and hospital admissions will drive further increases in imaging utilisation.^36^

## Imaging as a tool to understand climate health effects

Medical imaging can be leveraged as a non‐invasive tool to identify the adverse health effects of climate change, promote disease prevention and advance our understanding of the underlying mechanisms of climate‐related disease. For example, higher ultrasound‐derived carotid intima‐media thickness is associated with higher long‐term exposure to air pollution.^37^ Similarly, progressive atherosclerotic calcification characterised by CT is associated with higher long‐term exposure to fine particulate matter.^38^ In the acute setting, imaging is widely used to identify and characterise cardiovascular and respiratory diseases including acute myocardial infarction, stroke and chronic obstructive disease exacerbation, which can all be precipitated or exacerbated by climate‐related environmental exposures.^39^ Medical imaging can also be leveraged to promote health, prevent severe disease and reduce overall health care‐related greenhouse gas emissions. For example, coronary artery calcium score CT can be used to identify patients with subclinical coronary artery disease who might benefit from medical therapy, theoretically avoiding more costly and energy‐intensive future healthcare needs.^11^

## Climate change and health equity in radiology

Everyone is impacted by climate change. However, it does not affect everyone equally.^40^ The intersection between climate change and health equity is a dynamic and complex issue encompassing multiple social determinants of health including social, cultural, economic and political systems. Vulnerability to the effects of climate change is determined by increased exposure to climate and environmental hazards, increased susceptibility to damage caused by climate hazards and decreased ability to cope with and recover from the damage.^39^, ^41^ Individuals with more resources will be better equipped to adapt to a changing climate, to take actions to protect their health and to access healthcare and radiology services if needed.^42^

Globally, the greatest climate‐related health risks are among regions that already face health inequities, in populations that have contributed the least to climate change and often have the least means to adapt. Beyond the direct health impacts of climate change, high temperatures also lead to decreased labour productivity and income. For example, in 2022, Africa experienced the highest relative potential income loss as a result of heat exposure, which amounted to 4.1% of its GDP with 81% of potential income losses falling on the most vulnerable and least protected agriculture workers with the lowest incomes.^15^

At the individual level, climate change disproportionally affects marginalised individuals within a society, including those with preexisting health conditions and socioeconomically disadvantaged groups.^43^ These individuals are more likely to live in regions that are prone to environmental hazards, such as flood zones, heat islands, or areas with high pollution. This increased exposure exacerbates health risks related to climate change, such as respiratory problems, heat‐related illnesses and vector‐borne diseases. Additionally, these individuals often have less access to resources such as healthcare, reliable housing, food security and clean air and drinking water, resulting in higher susceptibility to the impacts of climate change. These disadvantages leave marginalised communities less equipped to adequately respond to natural disasters. This is evidenced by the aftermath of hurricane Katrina in New Orleans, LA, USA, where people with household income under $20,000 and those living with chronic illnesses were disproportionately impacted.^44^ Many living within these vulnerable communities were unable to seek shelter and acquire fresh food and clean water, adversely impacting their health.

Barriers to medical imaging also exist for those with lower socioeconomic status. Shah *et al*. 2020 investigated disparities in treatment for patients living with non‐small cell lung cancer in Ontario. In this study, patients with higher socioeconomic status were more likely to be imaged by MRI and subsequently were more likely to have lung resection surgery. Overall, they also had better 5‐year survival compared to those with lower socioeconomic status.^45^

Health equity and climate change impact radiology directly. The most vulnerable people and communities not only experience greater health effects of climate change, but they also have the lowest access to imaging services. Location and geography impact access to healthcare and imaging. Patients living in rural and remote areas may have limited access to specialty and emergency services and may need to travel greater distances to access care.^46^ For example, less than 20% of rural emergency departments in Canada have in‐house access to CT, necessitating patient transfers to other hospitals if CT imaging is needed.^47^ Climate‐related environmental and weather events can further disrupt and decrease healthcare and radiology access.^11^ In local and global health contexts, it is essential that capacity building in underserved areas occurs in parallel with mitigation strategies to reduce the environmental impact of the delivery of medical imaging. Further research is needed to characterise the impact of climate change on exacerbation of health inequities with respect to access to radiology services.

## Health‐centred response to climate change in radiology

Given wide‐ranging impacts of climate change on human health, delivery of low‐carbon and sustainable radiology services will promote human health through various means including reducing sources of emissions and addressing social inequities.^48^ Mitigation strategies may also have direct health co‐benefits, such as improved cardiovascular and respiratory health by promoting active transportation options or accessibility of plant‐forward meal options at work.^10^ Other benefits to human health include improved air quality, increased physical activity, healthier diets, reduced risk of infectious disease and improved access to healthcare.

**Fig. 2:**
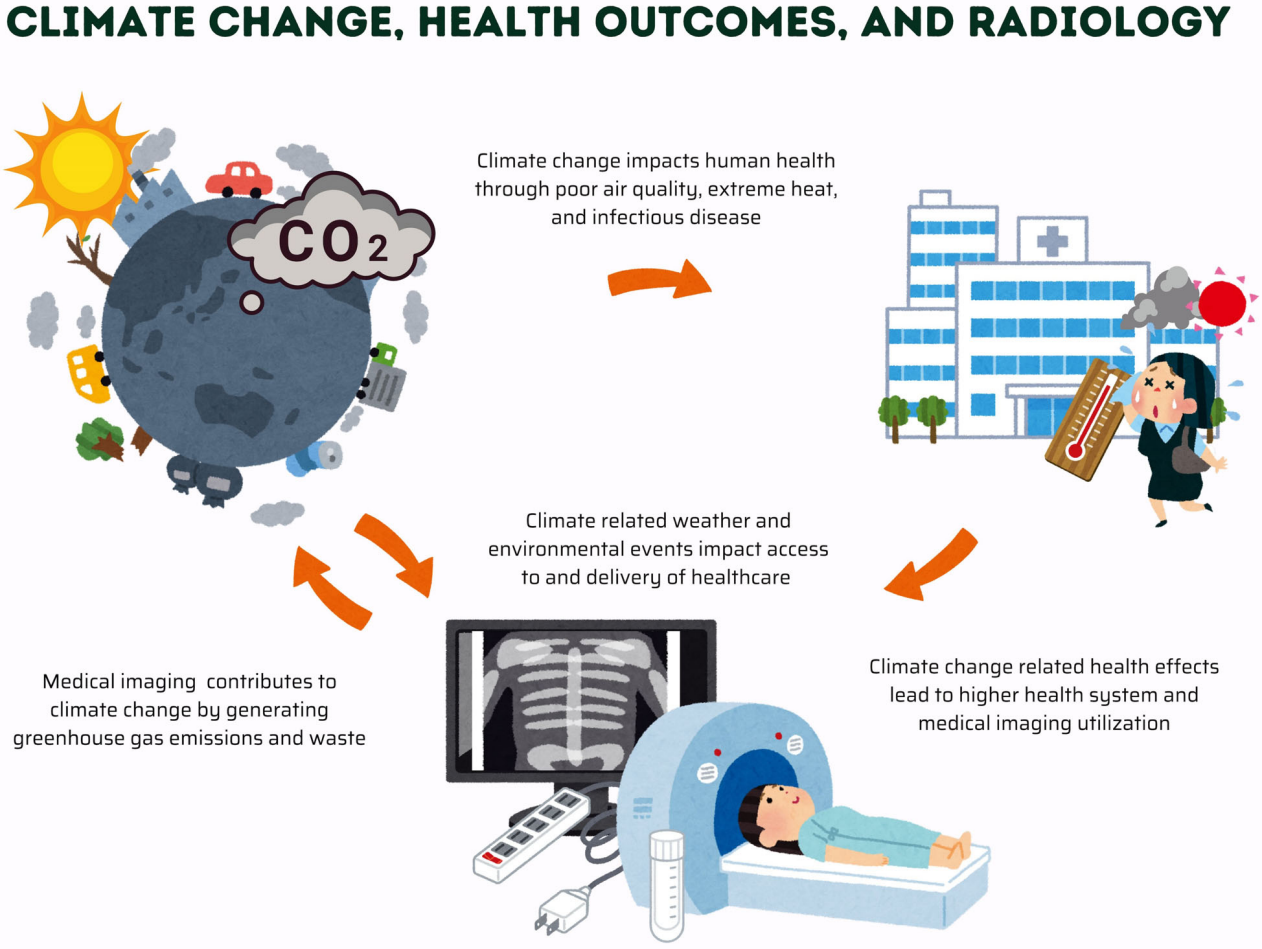
Interconnected relationships between climate change, health outcomes and radiology.

Mitigation strategies to reduce the environmental impact of radiology services have been described previously, including powering down medical imaging equipment when not in use, shortening protocols, reducing low‐value imaging and optimising waste management.^6^, ^8^, ^9^, ^12^ Technological innovations in radiology, including artificial intelligence tools, have the potential to further reduce energy use and associated greenhouse gas emissions.^9^ At the same time, radiology departments must also build resiliency to current and future impacts of the climate crisis.^10^ Adaptation strategies are needed to ensure that existing medical imaging equipment, infrastructure and workforce are prepared for the effects of climate change. Key actions include accelerating the development of climate‐resilient radiology systems and increasing the capacity of radiology departments to prepare for and respond to climate‐related health risks. The interconnected relationships between climate change, health outcomes and radiology are summarised in Figure 2.

**Fig. 3:**
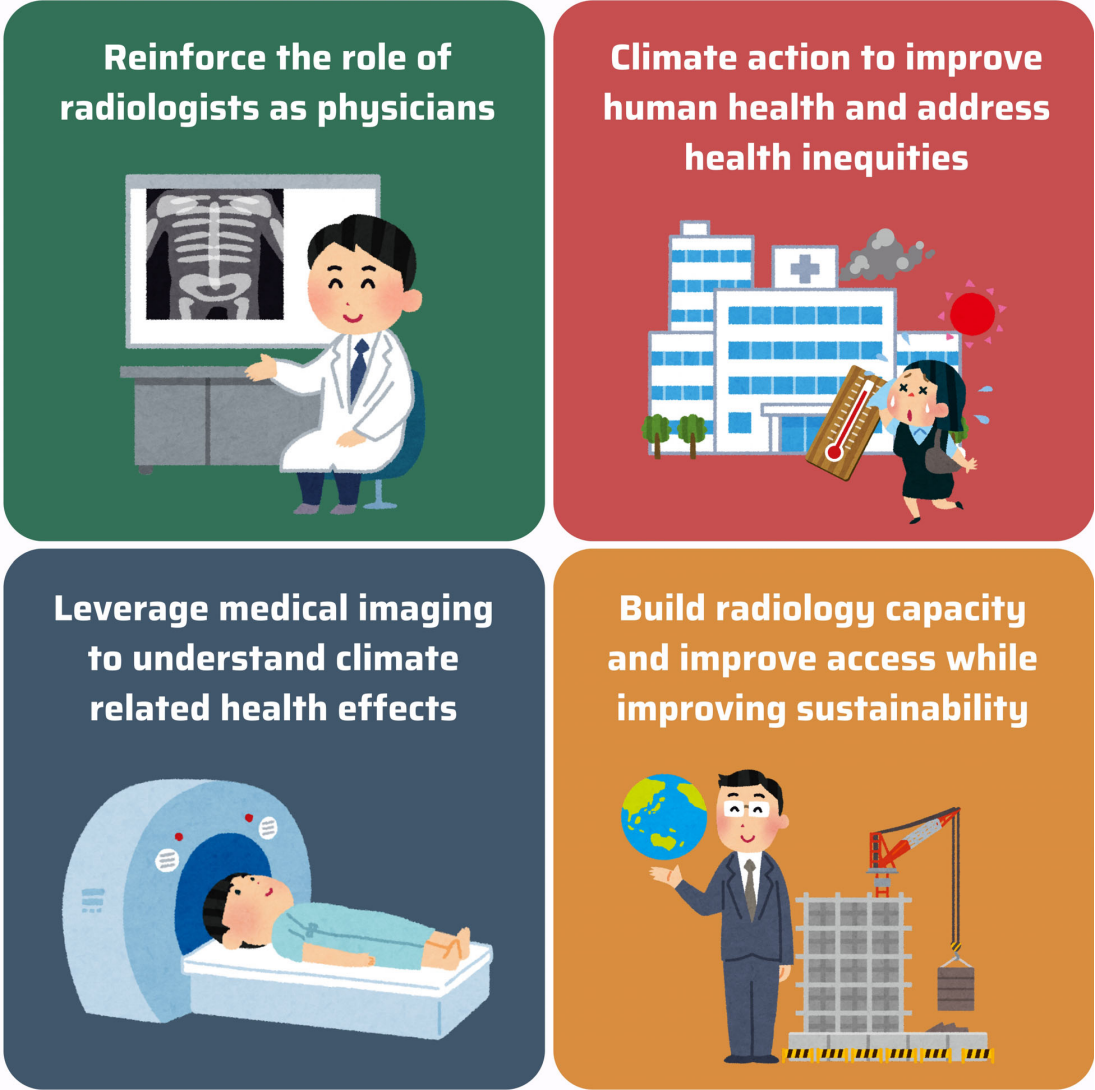
Actions and importance of a health‐centred response on climate change in radiology.

A health‐centred response in radiology reinforces the role of radiologists as physicians and emphasises the opportunity for medical imaging to promote health and advance our understanding of climate‐related health effects, Figure 3. This focus is essential to engage clinicians, administration teams, patients and other stakeholders in climate action. We all have a role to play in addressing the climate crisis and we must work together to deliver environmentally sustainable and climate‐resilient radiology services centred on human health.

## Conflict of interest

No conflicts of interest were declared.

## Data Availability

Data sharing not applicable to this article as no datasets were generated or analysed during the current study.

## References

[1] Crutzen PJ . The “Anthropocene”. In: Ehlers E , Krafft T (eds). Earth System Science in the Anthropocene. Springer, Heidelberg, 2006; 13–18.

[2] Myers SS . Planetary health: protecting human health on a rapidly changing planet. Lancet 2018; 390: 2860–2868. 10.1016/S0140-6736(17)32846-5 29146123

[3] Pongsiri MJ , Gatzweiler FW , Bassi AM , Haines A , Demassieux F . The need for a systems approach to planetary health. Lancet Planet Health 2017; 1: e257–e259. 29851620 10.1016/S2542-5196(17)30116-X

[4] Dzau VJ , Levine R , Barrett G , Witty A . Decarbonizing the U.S. health sector — a call to action. N Engl J Med 2021; 385: 2117–2119. 34644470 10.1056/NEJMp2115675

[5] Eckelman MJ , Sherman J . Environmental impacts of the U.S. health care system and effects on public health. PLoS One 2016; 11: e0157014. 27280706 10.1371/journal.pone.0157014 PMC4900601

[6] Brown M , Schoen JH , Gross J , Omary RA , Hanneman K . Climate change and radiology: impetus for change and a toolkit for action. Radiology 2023; 307: e230229. 37070994 10.1148/radiol.230229

[7] Heye T , Knoerl R , Wehrle T *et al*. The energy consumption of radiology: energy‐ and cost‐saving opportunities for CT and MRI operation. Radiology 2020; 295: 192084. 10.1148/radiol.2020192084 32208096

[8] Chaban YV , Vosshenrich J , McKee H *et al*. Environmental sustainability and MRI: challenges, opportunities, and a call for action. J Magn Reson Imaging 2024; 59: 1149–1167. 37694980 10.1002/jmri.28994 PMC11707703

[9] Doo FX , Vosshenrich J , Cook TS *et al*. Environmental sustainability and AI in radiology: a double‐edged sword. Radiology 2024; 310: e232030. 38411520 10.1148/radiol.232030 PMC10902597

[10] McKee H , Brown MJ , Kim HHR *et al*. Planetary health and radiology: why we should care and what we can do. Radiology 2024; 311: e240219. 38652030 10.1148/radiol.240219

[11] Gunasekaran S , Szava‐Kovats A , Battey T *et al*. Cardiovascular imaging, climate change, and environmental sustainability. Radiol Cardiothorac Imaging 2024; 6: e240135. 38900024 10.1148/ryct.240135 PMC11211952

[12] Ibrahim F , Cadour F , Campbell‐Washburn AE *et al*. Energy and greenhouse gas emission savings associated with implementation of an abbreviated cardiac MRI protocol. Radiology 2024; 311: e240588. 38652029 10.1148/radiol.240588 PMC11070609

[13] Hanneman K , McKee H , Nguyen ET , Panet H , Kielar A . Greenhouse gas emissions by diagnostic imaging modality in a hospital‐based radiology department. Can Assoc Radiol J 2024; 75: 950–953. 38742437 10.1177/08465371241253314

[14] Hanneman K , Szava‐Kovats A , Burbridge B *et al*. Canadian Association of Radiologists Statement on environmental sustainability in medical imaging. Can Assoc Radiol J 2024; 8465371241260013. doi: 10.1177/08465371241260013. 39080832

[15] Romanello M , Napoli C d , Green C *et al*. The 2023 report of the lancet countdown on health and climate change: the imperative for a health‐centred response in a world facing irreversible harms. Lancet 2023; 402: 2346–2394. 37977174 10.1016/S0140-6736(23)01859-7 PMC7616810

[16] Rocque RJ , Beaudoin C , Ndjaboue R *et al*. Health effects of climate change: an overview of systematic reviews. BMJ Open 2021; 11: e046333. 10.1136/bmjopen-2020-046333 PMC8191619 34108165

[17] Habibullah MS , Din BH , Tan SH , Zahid H . Impact of climate change on biodiversity loss: global evidence. Environ Sci Pollut Res 2022; 29: 1073–1086. 10.1007/s11356-021-15702-8 34341937

[18] Sandifer PA , Sutton‐Grier AE , Ward BP . Exploring connections among nature, biodiversity, ecosystem services, and human health and well‐being: opportunities to enhance health and biodiversity conservation. Ecosyst Serv 2015; 12: 1–15.

[19] World Health Organization . Ambient (outdoor) air pollution. 2022. [Cited 15 Aug 2024.] Available from URL: https://www.who.int/news‐room/fact‐sheets/detail/ambient‐(outdoor)‐air‐quality‐and‐health.

[20] World Heart Federation . World Heart Report 2024. 2024. [Cited 15 Aug 2024.] Available from URL: https://world‐heart‐federation.org/report2024/.

[21] Al‐Kindi SG , Brook RD , Biswal S , Rajagopalan S . Environmental determinants of cardiovascular disease: lessons learned from air pollution. Nat Rev Cardiol 2020; 17: 656–672. 32382149 10.1038/s41569-020-0371-2 PMC7492399

[22] Liang R , Zhang B , Zhao X , Ruan Y , Lian H , Fan Z . Effect of exposure to PM2.5 on blood pressure: a systematic review and meta‐analysis. J Hypertens 2014; 32: 2130–2140; discussion 2141. 25250520 10.1097/HJH.0000000000000342

[23] Bevan GH , Al‐Kindi SG , Brook RD , Münzel T , Rajagopalan S . Ambient air pollution and atherosclerosis: insights into dose, time, and mechanisms. Arterioscler Thromb Vasc Biol 2020; 41: 628–637. 33327745 10.1161/ATVBAHA.120.315219

[24] Moon J . The effect of the heatwave on the morbidity and mortality of diabetes patients; a meta‐analysis for the era of the climate crisis. Environ Res 2021; 195: 110762. 33515577 10.1016/j.envres.2021.110762

[25] Alahmad B , Khraishah H , Royé D *et al*. Associations between extreme temperatures and cardiovascular cause‐specific mortality: results from 27 countries. Circulation 2023; 147: 35–46. 36503273 10.1161/CIRCULATIONAHA.122.061832 PMC9794133

[26] Mora C , McKenzie T , Gaw IM *et al*. Over half of known human pathogenic diseases can be aggravated by climate change. Nat Clim Chang 2022; 12: 869–875. 35968032 10.1038/s41558-022-01426-1 PMC9362357

[27] George AM , Ansumana R , De SDK , Niyas VKM , Zumla A , Bockarie MJ . Climate change and the rising incidence of vector‐borne diseases globally. Int J Infect Dis 2024; 139: 143–145. 38096974 10.1016/j.ijid.2023.12.004

[28] MacFadden DR , McGough SF , Fisman D , Santillana M , Brownstein JS . Antibiotic resistance increases with local temperature. Nat Clim Chang 2018; 8: 510–514. 30369964 10.1038/s41558-018-0161-6 PMC6201249

[29] Ali KJ , Ehsan S , Tran A , Haugstetter M , Singh H . Diagnostic excellence in the context of climate change: a review. Am J Med 2024; 137: 1035–1041. 38925497 10.1016/j.amjmed.2024.06.010

[30] Tong MX , Wondmagegn BY , Xiang J *et al*. Emergency department visits and associated healthcare costs attributable to increasing temperature in the context of climate change in Perth, Western Australia, 2012–2019. Environ Res Lett 2021; 16: 065011.

[31] Lay CR , Mills D , Belova A *et al*. Emergency department visits and ambient temperature: evaluating the connection and projecting future outcomes. GeoHealth 2018; 2: 182–194. 32159014 10.1002/2018GH000129 PMC7007124

[32] Sorensen C , House JA , O'Dell K *et al*. Associations between wildfire‐related PM2.5 and intensive care unit admissions in the United States, 2006–2015. GeoHealth 2021; 5: e2021GH000385. 10.1029/2021GH000385 PMC8095362 33977181

[33] Doubleday A , Sheppard L , Austin E , Isaksen TB . Wildfire smoke exposure and emergency department visits in Washington state. Environ Res Health 2023; 1: 025006. 37252333 10.1088/2752-5309/acd3a1 PMC10213826

[34] Hanneman K , Taboun O , Kirpalani A *et al*. Increased emergency department medical imaging: association with short‐term exposures to ambient heat and particulate air pollution. Radiology 2024; 313: e241624. 39560481 10.1148/radiol.241624 PMC11605103

[35] Poyiadji N , Beauchamp N , Myers DT , Krupp S , Griffith B . Diagnostic imaging utilization in the emergency department: recent trends in volume and radiology work relative value units. J Am Coll Radiol 2023; 20: 1207–1214. 37543154 10.1016/j.jacr.2023.06.033

[36] Chrysanthopoulou A , Kalogeropoulos A , Terzis G *et al*. Trends and future needs in clinical radiology: insights from an academic medical center. Health Policy 2007; 80: 194–201. 16624441 10.1016/j.healthpol.2006.03.007

[37] Provost EB , Madhloum N , Panis LI , Boever PD , Nawrot TS . Carotid intima‐media thickness, a marker of subclinical atherosclerosis, and particulate air pollution exposure: the meta‐analytical evidence. PLoS One 2015; 10: e0127014. 25970426 10.1371/journal.pone.0127014 PMC4430520

[38] Rajagopalan S , Al‐Kindi SG , Brook RD . Air pollution and cardiovascular disease. J Am Coll Cardiol 2018; 72: 2054–2070. 30336830 10.1016/j.jacc.2018.07.099

[39] Hanneman K , Nguyen ET , Kielar A . Climate change, health equity, and environmentally sustainable radiology. Can Assoc Radiol J 2024; 75: 957. 39129214 10.1177/08465371241274183

[40] Deivanayagam TA , English S , Hickel J *et al*. Envisioning environmental equity: climate change, health, and racial justice. Lancet 2023; 402: 64–78. 37263280 10.1016/S0140-6736(23)00919-4 PMC10415673

[41] Islam N , Winkel J . Climate Change and Social Inequality. 2017.

[42] Schnitter R , Moores E , Berry P *et al*. Climate change and health equity. In: Berry P , Schnitter R (eds). Health of Canadians in a Changing Climate: Advancing our Knowledge for Action. Government of Canada, Ottawa, ON, 2022.

[43] Otto IM , Reckien D , Reyer CPO *et al*. Social vulnerability to climate change: a review of concepts and evidence. Reg Environ Change 2017; 17: 1651–1662.

[44] Benevolenza MA , DeRigne L . The impact of climate change and natural disasters on vulnerable populations: a systematic review of literature. J Hum Behav Soc Environ 2019; 29: 266–281.

[45] Shah M , Parmar A , Chan KKW . Socioeconomic disparity trends in diagnostic imaging, treatments, and survival for non‐small cell lung cancer 2007–2016. Cancer Med 2020; 9: 3407–3416. 32196964 10.1002/cam4.2978 PMC7221447

[46] Davidson M , Kielar A , Tonseth RP , Seland K , Harvie S , Hanneman K . The landscape of rural and remote radiology in Canada: opportunities and challenges. Can Assoc Radiol J 2023; 75: 304–312. 37638676 10.1177/08465371231197953

[47] Fleet R , Pelletier C , Marcoux J *et al*. Differences in access to services in rural emergency departments of Quebec and Ontario. PLoS One 2015; 10: e0123746. 25874948 10.1371/journal.pone.0123746 PMC4398492

[48] Mailloux NA , Henegan CP , Lsoto D *et al*. Climate solutions double as health interventions. Int J Environ Res Public Health 2021; 18: 13339. 34948948 10.3390/ijerph182413339 PMC8705042

